# ‘Big science’ in the field: experimenting with badgers and bovine TB, 1995–2015

**DOI:** 10.1007/s40656-015-0072-z

**Published:** 2015-07-04

**Authors:** Angela Cassidy

**Affiliations:** Department of History, King’s College London, Strand, London, WC2R 2LS UK

**Keywords:** Field science, RCTs, Science and policy, Evidence based policy, Agricultural research, Ecology, Animal health

## Abstract

Since wild badgers were first connected with outbreaks of bovine TB (bTB) in UK cattle herds in the early 1970s, the question of whether to cull them to control infections in cattle has been the subject of a protracted public and policy controversy. Following the recommendation of Prof. John Krebs that a “scientifically based experimental trial” be carried out to test the effectiveness of badger culling, the Randomised Badger Culling Trial (RBCT) was commissioned by Government in 1998. One of the largest field experiments ever conducted in the UK, the RBCT sought to recreate the conditions of a randomised controlled trial (RCT) across approximately 3000 km^2^ of the South West of England. Despite widespread expectations that the RBCT would provide the necessary evidence to resolve the controversy, its findings have instead been widely contested and reinterpreted, while arguments over badger culling have become increasingly polarised. This paper will investigate the complexities of field experimental knowledge by following the story of the RBCT from this initial proposal, through processes of research design, implementation, analysis, interpretation and reinterpretation of the findings by multiple actors. It asks what kind of experiment the RBCT actually was, and examines how it has contributed to the protracted controversy over whether to cull badgers in order to control bTB in cattle. Finally, it will explore the wider implications of this case for contemporary debates over the contribution that RCTs can make to formulating public policy.

## A ‘proper experiment’

On 23rd July 1996, the UK Secretary of State for Agriculture, Douglas Hogg, announced an independent scientific review into British government policy on “TB in cattle and badgers” (HC Deb 23 July 1996 vol. 282 c262W). The enquiry was chaired by Professor (now Lord) John Krebs, and was the third such expert review since 1980: a pattern which has continued subsequently, with three further groups of ‘independent experts’ having been called upon by successive UK governments to the present day. Krebs’ review group published their report in late 1997 (Krebs et al. 1997). They concluded that while the evidence gathered since the early 1970s by the UK’s Ministry of Agriculture, Fisheries and Food (MAFF) research programme on badgers and bTB supported the idea that bTB infections in wild badgers were being passed into domestic cattle herds, further scientific investigation was required. The Krebs group recommended conducting a major field experiment in order to directly test the effects of badger culling on TB rates in cattle. Their rationale was that while existing research on the topic had provided a great deal of information about the issue, none of it had involved a ‘proper experiment’, meaning a study comparing experimental interventions with a control condition without intervention, where the application of each condition is randomly applied. They argued that such a study would provide high-quality evidence of the effects of badger culling, and as such be able to directly shape policy formation on TB control:

A proper experimental assessment is the only way to test rigorously the effectiveness (and cost-effectiveness) of different strategies and to provide a sound basis for future policy. Although this would have significant resource implications for Government, these must be considered in the context of the actual and potential costs of TB. An analogy might be the evidence required to recommend the widespread use of a new therapeutic drug. (Krebs et al. 1997, p. 128)

At this point in time badgers and bTB was already a notoriously fraught problem which had remained unresolved for over 20 years, despite extensive research efforts. Krebs’ arguments were convincing to the incoming Labour administration, and in 1998 they commissioned a new Independent Scientific Group (ISG) to design and implement the proposed experiment. The Randomised Badger Culling Trial (or RBCT), is probably the largest scale field trial yet conducted in the UK, certainly in the sphere of animal health. It was carried out across over 3000 km^2^ of the South West of England, took over 9 years to complete, culled 10,979 badgers, and cost just over £49 million (Bourne et al. 2007; Defra 2011). Since it ended in 2007, the findings and conclusions of the RBCT trial have been cited, presented, interpreted, contested, analysed, re-analysed and re-interpreted by multiple scientific, veterinary, political, policy, public and campaigning actors. They have argued for and against badger culling across journals, policy reports, Parliamentary debates and committees, and the British mass media in a highly polarised, ongoing public controversy (Cassidy 2012; Lodge and Matus 2014).

This article will follow the story of the RBCT, starting with the scientific, political and policy contexts of Krebs’ proposal, through the processes of research design, implementation in the field, to the analysis, interpretation and reinterpretation of these findings once they were made public. By doing so, it will address a series of questions about the roles of science, evidence and expertise in the recent history of bovine TB (bTB) in the UK. It will centre these questions on the above quotation from Krebs, asking *what kind of experiment* was the RBCT? What was meant when the group wrote of ‘a proper experiment’, and what did this imply about the research which had already been done? How was the experiment implemented in practice, and did it achieve its goals? While the relationship between science and policy is rarely straightforward, was there anything about ‘the science’ of the RBCT which particularly contributed to this process of contestation?

In order to answer these questions, this article will draw upon the varying histories of the randomised controlled trial (RCT), as developed and implemented in the medical (Edwards 2007), agricultural (Berry 2015) and behavioural sciences (Hacking 1988). It will bring this into dialogue with research addressing the particular challenges of doing science beyond the relatively easily controllable conditions of the laboratory. This includes work on designing field experiments in agricultural research (Henke 2000); field epidemiology (Amsterdamska 2005; Steere-Williams 2015), and on field investigations of natural environments and animal behaviour (Burkhardt 1999; Gay 2013; Kohler 2002; Rees 2009). While this article will focus on the RBCT rather than the wider public debate about badgers and bTB, literature exploring agricultural knowledge production and expertise in the relations of scientists, other professions, government, industry and wider publics is also of key relevance (Harwood 2012; Woods 2011, 2014), particularly in cases of controversial and contested science (Bonneuil et al. 2007). By answering these questions, this article can also contribute to current debates ranging far beyond the history and philosophy of science about the proper relationship between science, ‘evidence’ and policymaking (Cartwright and Hardie 2012). RCTs have formed a significant focus for this debate in the UK, with recent governmental and scientific actors controversially advancing arguments that they should be used more frequently to determine policy (Pearce and Raman 2014). Given that the RBCT is one of the largest and most high profile examples of such an experimental intervention in policy in the UK, this recent history is likely to provide valuable lessons to inform these ongoing debates.

## Bovine tuberculosis: a chronic science/policy problem for the UK

Bovine tuberculosis (bTB) is an infectious disease caused by *Mycobacterium bovis*, primarily affecting domestic cattle, but with the potential to infect most mammals including humans. Historically in the UK it was an important source of clinical disease, particularly in children, and it remains a significant global health problem (Michel et al. 2010). For this reason, transmission of the disease to humans has been controlled in higher income countries since the mid-twentieth century via a combination of milk pasteurisation; meat inspection; cattle movement controls, and testing/slaughter regimes (Atkins 2010; Hardy 2010; Waddington 2006). By 1960, eradication was confidently predicted: however over the following decades the infection rate started to slowly increase, and then rose sharply from the early 2000s (Defra 2014, p. 24). In 1971, when a dead badger was found on a farm in Gloucestershire suffering persistent outbreaks of bTB, MAFF veterinarians directly connected TB infections in wild badgers with cattle disease (Muirhead et al. 1974). Shortly afterwards an Act of Parliament was passed protecting the animals from being killed or interfered with, which also put into place a legal framework for licensing ‘badger control’ (culling) activities. By 1975 a policy of culling badgers in response to TB outbreaks in cattle was in place, alongside a rapidly developing programme of research into the links between cattle, TB and badgers, including disease mapping; pathological and microbiological investigations; field research into badger ecology and behaviour; and ‘clearance’ (culling) studies.^1^ At the end of the decade the eminent zoologist and government scientist Lord Solly Zuckerman was asked to review the situation. He endorsed existing policy, but precipitated further investigations into the methods used (gassing setts with cyanide), which were subsequently banned (Zuckerman 1980).^2^

The 1980s saw further expansion of the MAFF TB research programmes, a second scientific review (Dunnett 1986), and several adjustments to culling policies. In the meantime, TB rates in cattle continued to climb, and it was in this context, alongside the political pressure on agriculture and animal health engendered by the BSE crisis, plus an impending General Election, that Agriculture Secretary Douglas Hogg commissioned John Krebs to lead yet another expert review on the subject. Krebs rapidly assembled an Independent Scientific Review Group, consisting of himself and a multidisciplinary group including epidemiologist Roy Anderson, two zoologists, an immunologist, a microbiologist and a statistician. Their report, delivered in 1997, concluded that the existing evidence “strongly supports the view that badgers are a significant source of infection in cattle” (Krebs et al. 1997, p. 6).^3^ However, they also highlighted the uncertainties around the studies of badgers, cattle and TB that had been done to date, arguing that while this body of work provided many important insights into the problem, much of the evidence it provided was ‘circumstantial’ because it relied upon correlations or single interventions. Similarly, the various culling policies that had been implemented had involved differing scales, local geographical features, quantities, frequencies and timings of culls, making it impossible to compare them with each other, or with a lack of intervention. The Krebs group argued that a new approach was required, and that a new experiment be conducted to directly test the effects of badger culling on TB rates in cattle. They proposed a randomised design comparing three experimental conditions: culling badgers *reactively* in response to TB ‘breakdowns’ (herd level infections) in cattle, culling *proactively* before breakdowns occurred, and a *control* condition where information was gathered but nothing else was done. This should be carried out in areas where bTB was particularly prevalent, and all other culling policies should be suspended. Krebs et al. proposed that, unlike the earlier research, which had largely been directed and conducted by MAFF’s own scientists, this study should be designed and implemented by another ‘Independent Expert Group’, who would monitor and analyse the findings (it was envisaged that the study would be complete within 5 years). Finally, they envisaged that their proposed experiment would “provide unambiguous evidence on the role of the badger in cattle TB”, and that “it would provide a basis for determining appropriate policies” for controlling bTB (Krebs et al. 1997, p. 128).

## ‘Big science’ in the field: implementing the experiment

Following their election victory in May 1997, Labour’s new Secretary of State for Agriculture, Jack Cunningham, took delivery of the Krebs report that summer, approving the formation of a new ISG in early 1998 to implement the new scientific strategy.^4^ As before, the new ISG was a multidisciplinary group of senior scientists, three of whom had been involved in the Krebs group (Krebs himself moved on to head the newly formed Food Standards Agency). It was chaired by John Bourne, a veterinarian and professor of animal health, and was initially composed of Bourne, plus three statisticians, an immunologist and a mammal ecologist. Several months later, the ISG invited John McInerney, an agricultural economist, to join the group. Formally, they were charged with the design, implementation and monitoring of the randomised trial, but from their first report they robustly claimed a wider remit: “to recommend a combination of measures which, taken together, will provide information essential for the establishment of future policy” (Bourne et al. 1998, p. 3).

**Fig. 1:**
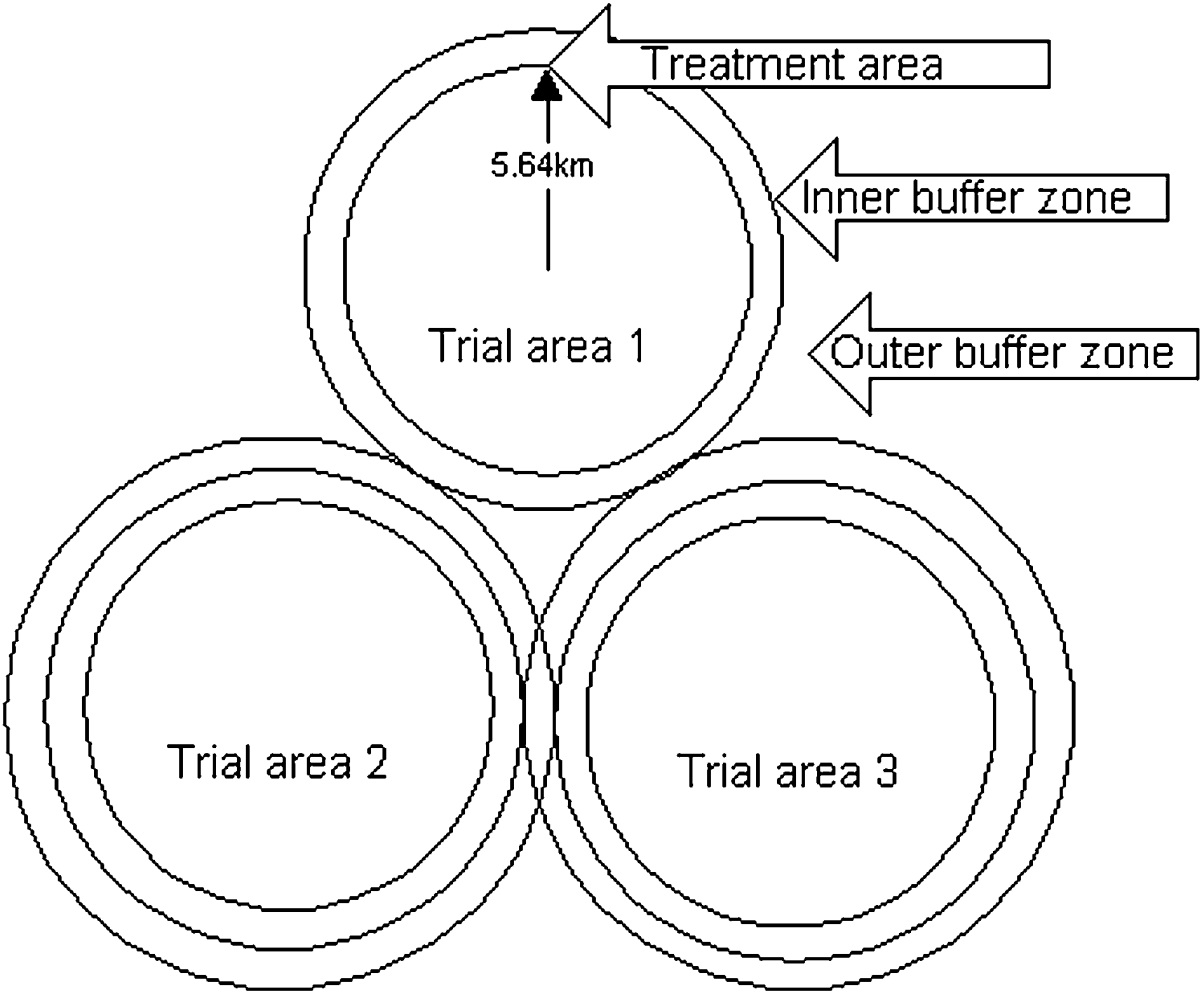
Envisaged ‘triplet’ of 100 km^2^ areas for testing badger culling (Bourne et al. 1998, 4.18; © Crown copyright 2015)

Once formed, the ISG drew up a priority list of the scientific questions to be addressed: these included questions of cross-species disease transmission, wildlife ecology, molecular disease typing, and examining alternative TB control strategies: as such the ISG oversaw a wider research programme alongside the core field trial. The trial itself was designed in line with Krebs’ recommendations: comparing the effects of reactive and proactive culling against each other as well as against a ‘control’ condition. The ISG decided that these conditions would be implemented across 10 ‘triplet’ areas (see Fig. 1)—sets of three adjacent circles of land, each with an approximate area of 100 km^2^, rather than the 10 × 10 km^2^ Krebs had envisaged. However, the overall scope of the planned experiment remained unchanged (a total field trial area of 3000 km^2^) and it retained the crucial concept of randomly assigning the experimental conditions to the experimental areas.

The ISG made a second set of early modifications to the original design. The practicalities of surveying potential study areas, negotiating with landowners across the South West of England, responding to MAFF’s formal public consultation, and waiting for Ministerial approval took some time. These delays meant that instead of implementing the experimental design across all 10 ‘triplet’ areas at once, it was put into place gradually as different areas became available. Krebs had also emphasised the need to remove *all* badgers in areas assigned to the culling conditions, including repeating culls to prevent ‘recolonisation’ (Krebs et al. 1997, p. 128). This aspect of the plans had come under severe criticism from wildlife and animal welfare groups, and the ISG took care to ensure their methods were as humane as possible, employing the cage-trapping/shooting procedure developed by MAFF since the 1980s. The ISG also modified the design to take account of concerns that lactating sow badgers would be killed, leaving their cubs to die of starvation underground. Instead of working all year round, they announced the use of a ‘closed season’ whereby animals would not be culled during the badger breeding season of 1 February–30 April. This was not simply a matter of squeamishness, convention or politics: as a signatory to the above-mentioned Bern Convention, any government action involving killing wildlife must be done as ‘humanely’ as possible, shaping the decisionmaking process (Bourne et al. 1998).

**Fig. 2:**
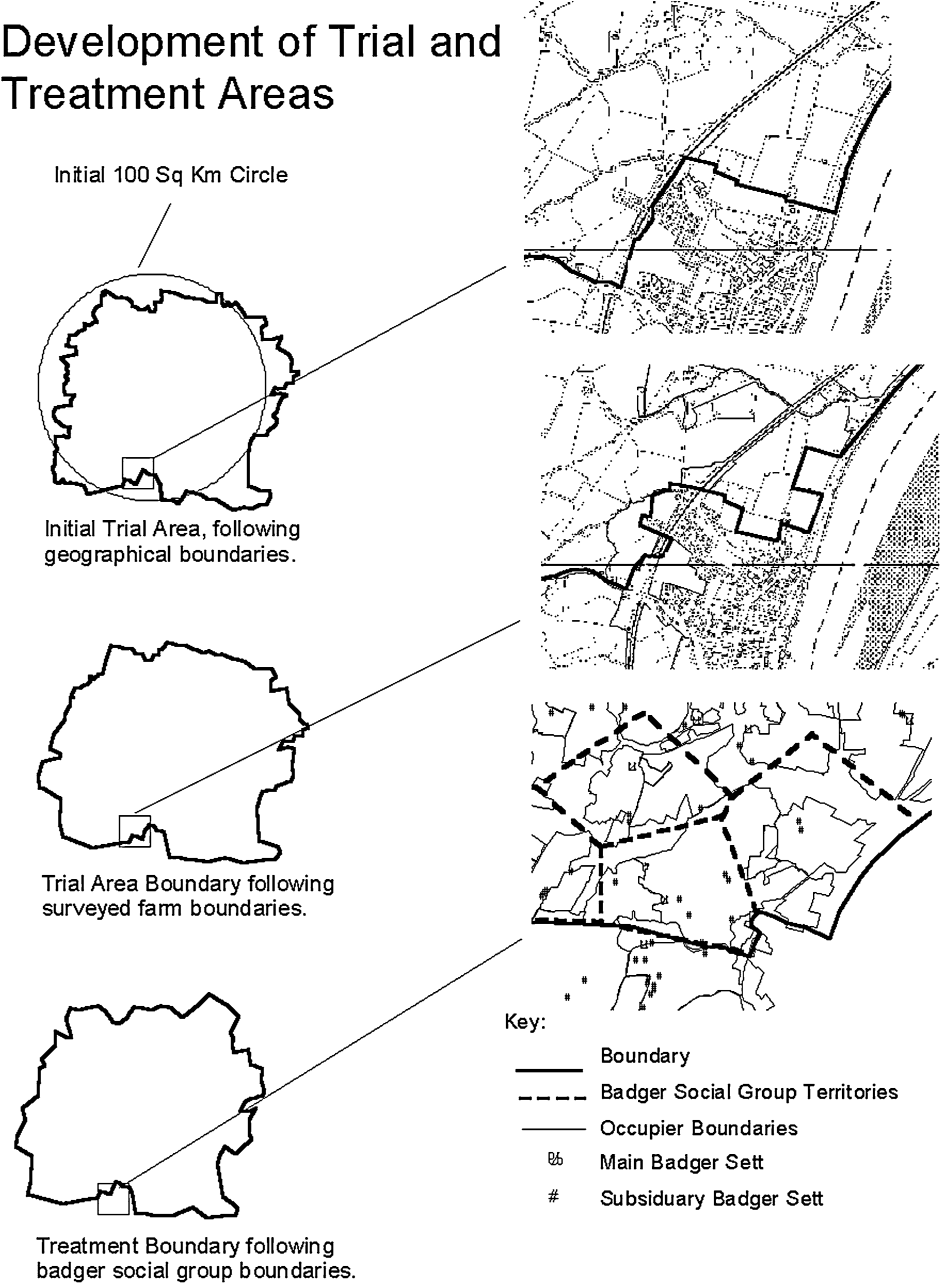
Translating experimental design to landscape (from Bourne et al. 1999, p. 27; © Crown copyright 2015)

By a year into the trial, the ISG were working on the complex job of translating the envisaged ‘triplet’ design across the landscapes of the SW of England, taking account of both human and badger social organisation (Fig. 2).

They reported a range of complications with surveying potential trial areas, gaining consent from landowners, recruiting staff to undertake culling and other fieldwork, and localised ‘interference’ with staff and equipment (Bourne et al. 1999). This was a reference to the direct action taken by badger and animal liberation activists, involving destroying cage-traps, releasing animals, harassing field staff and damaging equipment (CBAG 2014). These protestors continued these activities as an ongoing campaign throughout the RBCT, and similar tactics have been employed to disrupt more recent culling trials (BBC 2014), alongside widespread public campaigning (Lodge and Matus 2014).

In 2001 the complex work of implementation became even more difficult when the UK suffered its first epizootic of foot and mouth disease (FMD) since 1967. By this time, the ISG had surveyed all ten of the proposed triplet areas and started culling operations in seven of these, although one had been suspended due to ‘significant levels of interference’ (Bourne et al. 2001, p. 16). The severity of the FMD outbreak was such that all meat exports to the EU were suspended, as were animal and human movements in the affected areas. By the end of the outbreak between 6.5 and 10 million cattle were slaughtered, with long term economic and psychosocial impacts on farming communities (Convery et al. 2008). It also precipitated further reform of MAFF, removing food regulation to a separate Food Standards Agency and reorganising the ministry into the Department of the Environment and Rural Affairs (DEFRA). For the following year all ISG field investigations were suspended, largely because all the personnel involved were seconded into the FMD control effort (EFRAC 2002/2003, p. 83). When the ISG fieldwork was resumed, it became clear that this suspension would have a knock on effect, because the field aspects of MAFF’s routine bTB control procedures, including herd surveillance, testing, movement restrictions and cattle slaughter operations were also suspended during 2001. When these resumed in January 2002, it rapidly became clear that bTB incidence in cattle had risen sharply, renewed enquiries in Parliament and increased pressure from farming and veterinary groups for new policy action, rather than continuing with the ISG’s research strategy (EFRAC 2003/2004, p. 6).

The reactive cull condition suffered from particularly severe delays during the FMD outbreak, followed by a further backlogs as TB testing resumed (Bourne et al. 2005, p. 23). Once the ISG’s fieldwork finally resumed and the preliminary data was analysed the following year, an unexpected finding emerged, which the ISG advised ministers of immediately:

…the incidence of herd breakdowns in reactively culled areas has been consistently greater than expected. This increase was estimated to be 27%, though it could be as small as 4.3% or as large as 53%. This increase was consistent in all nine triplets that had received reactive culls by the time of analysis (triplet J has not yet been reactively culled). While the larger adverse effects may be implausible on general grounds, even a 10% deterioration, if it persisted, would clearly be of major concern. (ISG advice to DEFRA, 29th October 2003, quoted in Bourne et al. 2005, p. 120)

Not only was reactive culling not reducing TB rates in cattle, these early findings suggested that it was actually making the situation worse. The ISG recommended continuing to collect data for that season, then to stop the experimental condition as it was “not a viable base for a future policy option” (Bourne et al. 2005, p. 121). Instead ministers decided that swifter action was required, and the junior Minister at the time, Ben Bradshaw, announced the immediate suspension of the ‘reactive’ treatment areas of the field trial (Defra 2003). Alongside this announcement, the ISG’s initial analysis was published as a letter to *Nature* (Donnelly et al. 2003), alongside public statements explaining the situation (e.g. Elliot 2003). It was during this fourth report, published early in 2005, that the ISG started formally referring to the experiment as the ‘Randomised Badger Culling Trial’ or RBCT.

## Perturbing findings, policy recommendations

**Fig. 3:**
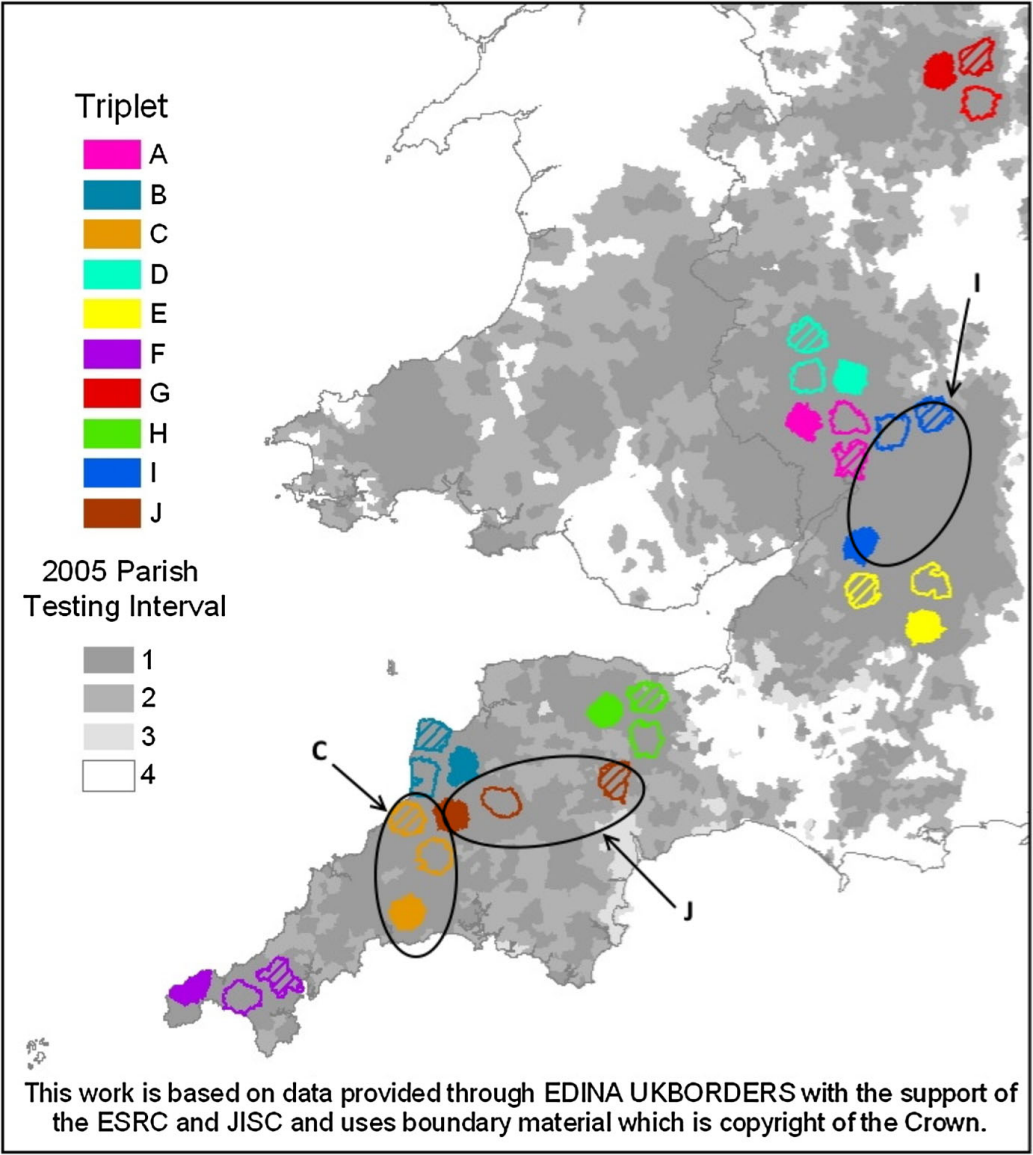
RBCT experimental conditions as implemented across the geography of the South West of England—note triplets ‘C’, ‘I’ and ‘J’ (from Bourne et al. 2007, p. 46, originally published in Donnelly et al. (2006); © Crown copyright 2015, also reprinted by permission from Macmillan Publishers Ltd: Nature, Donnelly et al 2006)

For the next few years, the ISG concentrated on completing the remaining parts of the experiment and publishing their findings in scientific journals: by the end of the trial they had published ten further research articles, plus various reviews, reports and conference papers (Bourne et al. 2007, pp. 243–251). Their next report, the fifth in the series (Bourne et al. 2006) was focused on this publication strategy, using it as a jumping off point to discuss their initial findings on the ‘proactive’ culling condition. These findings seemed complex and contradictory: while they found that TB incidence was 19 % *lower* where badgers had been proactively culled, it was 29 % *higher* in the area surrounding these cull zones (Bourne et al. 2006). This received relatively little public attention until the ISG published their Final Report on the 18th June 2007. This 289 page document, published 10 years after Krebs’s initial report, laid out the full findings and conclusions of the ISG, drawn from the RBCT trial as well as their broader research effort. Figure 3 demonstrates the final scale of the completed experiment, alongside its detailed positioning across the South West of England. Despite the early abandonment of some trial areas and of the reactive condition, the ISG did complete all ten of the planned ‘triplets’, although as can be seen, some of them ended up nowhere near as adjacent as had originally been envisaged. The field operations of the ISG ran from June 1998 until October 2005—a period of 7 years, while from inception to final report, the ISG’s research investigation took over 9 years to complete.

In this report the ISG repeated their key findings: that *reactive* culling increased bTB incidence, while *proactive* culling decreased it in the areas where badgers were culled, but made the problem worse nearby. They argued that the RBCT data showed that bTB was transmitted between badgers and cattle, but that cattle-to-cattle transmission also contributed to the spread of the disease. Finally, drawing on the mathematical and economic aspects of their work, they reported the findings of cost–benefit analysis indicating that culling badgers was simply not worth the investment of time and money required. The ISG had also developed a coherent theoretical explanation for these apparently contradictory findings: the perturbation effect:

The disruption of the social organisation or structure of badger populations, such as that which is caused where trapping/culling has taken place (Bourne et al. 2007, p. 288)

Badgers are omnivorous, nocturnal, digging animals, which in the UK tend to live in unusually large family groups defending well defined, long term territories (Roper 2010). The ISG argued that badger culling, particularly when undertaken sporadically, disrupts this social and spatial organisation, leading to individuals moving around more than they would otherwise and spreading bTB as they do so. The scientific term ‘perturbation’ is used throughout the sciences to indicate unusual patterns of interaction or movements. It has been used in ecology since the 1970s, describing the consequences of ecosystem disturbance (e.g. Loya 1976), and had been further developed in research on animal territoriality. Mammal ecologists had already linked perturbation effects to the spread of disease in the case of rabies in foxes (Doncaster and Macdonald 1997), and some badger campaigners had long speculated that culling and hunting the animals could spread bTB (Overend 1980). The idea that perturbation of badger territories might affect the spread of bTB had been suggested by modelling work prior to the RBCT (Swinton et al. 1997), and had been investigated as a side-study early in the experiment (Tuyttens et al. 2000).

However, it would seem that the strength of the perturbation effect on the spread of bTB had not been anticipated even by the ISG, and it was not until their final report in 2007 that they explained the idea clearly for nonscientific audiences:

Proactive culling substantially reduced badger population density, both on culled land and on nearby land that was either inaccessible for culling or outside the culling area. This density reduction was associated with disruption of badgers’ territorial system: badgers ranged more widely, and substantial numbers immigrated into the culled areas from neighbouring lands. Probably as a result of this perturbation, *M. bovis* prevalence in badgers rose substantially in response to culling, and infection became more diffuse across the landscape. (Bourne et al. 2007, p. 85).

The ISG argued that perturbation explained both the acute increases observed in cattle TB following ‘reactive’ culling, and the ‘edge effect’—increases in the areas surrounding those that had been ‘proactively’ culled. They also made a series of policy recommendations based on their findings. Their central recommendation was emphatic and relatively simple: that tightening the existing regulatory framework of TB testing and movement control of cattle herds was the most viable policy, and that “…badger culling cannot meaningfully contribute to the future control of cattle TB in Britain.” (Bourne et al. 2007, p. 14). Four members of the ISG gave evidence to a Parliamentary select committee enquiry on bTB on the day that their final report was published (EFRAC 2007, p. 63), and John Bourne gave a series of media interviews, ensuring that the ISG’s findings circulated widely.

## Cutting the cake of science and policy: the aftermath of the ISG

The ISG came under immediate criticism from representatives of the NFU, Conservative politicians and commentators, who renewed public calls for a badger cull (Clover 2007), while government responses to the report were muted, stating only that they would ‘consider carefully’ the ISG’s conclusions. Four months later, the government’s Chief Scientific Adviser, Sir David King, published his own report on ‘Bovine Tuberculosis in Cattle and Badgers’ (King et al. 2007). King, a chemist known for his interventions in public debates over climate change, also worked with an expert group: a mammal ecologist, an immunologist specialising in human TB, and three veterinary scientists (specialists in epidemiology, veterinary public health, and microbiology): their position was endorsed by the Chief Veterinary Officer. King’s report was a direct rebuttal of the ISG, concluding that culling in high incidence areas was the “best option available at the moment to reduce the reservoir of infection in wildlife” (King et al. 2007, p. 4). Given that they drew upon the ISG’s own data, how did the King group reach this very different conclusion? The ISG had taken on a deliberately broad remit, integrating scientific questions with policy considerations: they had described the RBCT as “a trial of potential culling policy options” (Bourne et al. 2007, p. 38). By contrast, King’s group only considered the effects of ‘badger removal’, without weighing this against alternative policy actions such as cattle controls and vaccinations. They also “focused on the scientific basis” (p. 3), thereby excluding considerations of animal welfare, the practicalities of culling methods, and the economic implications, including the cost–benefit analysis conducted by the ISG. King’s group considered in isolation the ISG’s figures on proactive culling, and the variance in that data, including estimates of how many animals needed to be killed and how large an area was necessary. They combined these figures with mathematical modelling, expanding the potential area to be culled, using ‘hard’ boundaries such as rivers or major roads to prevent badgers from moving around, and considered the effects of sustaining culling over a longer period of time.

The ISG scientists publicly and immediately defended their claims while pointing out that King’s conclusions were not applicable to realistic field or policy conditions. Over the autumn of 2007, the ISG, King, politicians and other actors campaigning for and against badger culling engaged in an extraordinary public controversy, fought in the mass media as well as the reports and committee enquiry rooms of the House of Commons, as reported later by an MP:

The scientists—Professor John Bourne, Christl Donnelly, Rosie Woodroffe and Sir David King—gave evidence before us. The atmosphere between them was interesting; it was probably more of an atmosphere than we sometimes have in here for Prime Minister’s Question Time, such was their commitment to the work they had done. (HC Deb 25 Oct 2012, vol 551, col. 1148)^5^

Further critiques emerged, this time from ex-government veterinarians and veterinary epidemiologists. These tended to focus on the design and implementation of the RBCT, in particular the adjustments that the ISG had made to adapt to policy, welfare and practical requirements, the curtailment of the ‘reactive’ condition, and the effects of direct action on field operations. The vets argued that this had meant not enough badgers had been removed from the trial areas, increasing the disruption to their social groups. This resulted in a flawed experiment which could not support the ISG’s claims; some argued that the design of the RBCT was such that it would have actually induced perturbation and was not comparable to a full culling policy (e.g. EFRAC 2007, ev29, 164, 172; Godfray et al. 2004; Sainsbury and Gallagher 2007; More 2009).

As the still-ongoing Parliamentary enquiry continued, it unravelled the ‘behind-the-scenes’ sequence of events (EFRAC 2007a, pp. 21–25), which sheds considerable light on the actions of all concerned. As their work approached completion, the ISG had sent ministers a ‘near to final’ draft of the report on 23rd May, but received little or no feedback. It would appear that as the ISG’s findings about perturbation had emerged over the previous few years, the relationship between DEFRA, ministers and the ISG had gradually deteriorated. Newspaper reports had suggested that Prime Minister Tony Blair and Defra Secretary David Miliband were preparing to implement a culling policy prior to the completion of the RBCT (e.g. Woolf 2005), even up to a few weeks prior to the publication of the ISG’s report (Cleland 2007).^6^ The EFRAC committee also discovered that shortly after the publication of the ISG’s final report, the DEFRA Secretary of State (then David Milliband) contacted Sir David King and asked him to “…undertake a short objective assessment of the key scientific issues”, which “did not extend to economic or other practical issues” (EFRAC 2007b, Ev 83). The report was delivered to ministers 6 weeks later, but was not made public until October 2007, precipitating the public controversy. It was not until October 2007 that ministers met directly with the scientists of the ISG to discuss their findings.

The lack of engagement between the ISG and ministers was also due to political upheaval within government at the time: on the 27th June, only 10 days after the ISG’s report, Gordon Brown took over from Tony Blair as Prime Minister, and as part of this power shift within the Labour administration, a new Defra Secretary, Hilary Benn, was appointed. It was therefore Benn who took delivery of the King report, releasing it to the public in October and then meeting the ISG scientists. Defra had run a consultation on badger culling during 2006, in which they had asked for public opinions on whether to cull in the first place, and their preferences over how to deliver such a policy. The consultation responses had come back with 95.6 % against culling, in part due to effective public campaigning from wildlife and animal welfare NGOs (Defra 2006). However, it would seem that these findings did not have much influence on policy until the Brown administration took over. In July 2008, Benn announced that “after a great deal of consideration”, he had decided not to implement a culling policy, and to instead invest £20 million on a new programme of bTB vaccination research, a decision based primarily on the advice of the ISG (BBC 2008). While the announcement was welcome by badger groups, conservationists and animal welfare campaigners, it produced a vehement reaction from pro-cull actors: the Minister was rapidly nicknamed ‘Veggie Benn’ in the rightwing press. From this time onwards, both sides of the controversy developed a common rhetoric, arguing that ‘sound science’ or ‘the evidence’ supported their arguments, while selectively drawing on different scientists (the ISG or King) to do so (Macrae 2007; Derbyshire 2008).

Benn’s policy was short-lived, as the Brown administration went on to lose the next General Election in 2010 to a coalition government composed of the Conservative and Liberal Democrat parties, who had both campaigned with pro-cull positions. The Coalition’s policy, involving culling badgers using a ‘free shooting’ method has been highly controversial, leading to increased public attention and polarisation of the debate. This policy has been supported in part by a detailed reanalysis of the RBCT data, involving the same strategy of excluding economics and finding conditions under which culling might work, as well as bringing in new data on the longer term effects of the RBCT (Defra 2014). Over the same period Welsh policy has turned about from a nationalist-led pro-culling policy, adopted during 2008, to a Labour-led vaccination and cattle control policy. Due to the devolution of agricultural policy to Wales, Scotland and N. Ireland, we have now seen six different bTB policies enacted across the UK since the completion of the RBCT, all built upon largely the same evidence base. Policy shifts around badgers and TB have been associated with shifts in political power since at least the post-Thatcher appointment of Lord Zuckerman. However, this more dramatic pattern—of turnabouts in policy supported by careful re-interpretations of data, rhetorically described as ‘the science’—appears to have been set during the 2007 controversy over the RBCT.

## Discussion

So what kind of experiment was the RBCT? When making their case, Krebs et al. (1997) invoked the RCTs of contemporary biomedicine, and certainly the RBCT shared key aspects its research design with RCTs. The RBCT involved directly comparing the effects of two different interventions with a control case, and doing so multiple times, just as would be done to test new drug treatments. Further parallels can be seen in the usage of preliminary assessment and consent procedures, alongside randomisation for deciding which particular areas would undergo which experimental intervention.^7^ However, looking beyond these central elements, Krebs’ comparison with evidence based medicine starts to disintegrate. Contemporary biomedical research generally involves the collation and comparison of the findings of hundreds, sometimes thousands of RCTs. In this case, there was only one RBCT, and given the time and cost involved, it seems unlikely that another such trial will be carried out in the foreseeable future. While clinical trials generally use a placebo intervention in their control conditions to avoid participants knowing whether or not they are receiving a genuine drug, it was impossible for the RBCT to do this. While the ‘participants’ were in this case inanimate patches of land, they were at the same time far more animate rural ecologies, including animal and human communities well aware of whether culling was taking place or not and reacting accordingly, actively resisting the experiment at times. Unlike most biomedical research, the RBCT was not carried out in the relatively controllable spaces of the lab or the clinic, but across the varied and complex landscapes of the South West of England. As an experiment conducted largely on farms, ultimately with the aim of improving the production of milk and beef, strictly speaking the RBCT was an agricultural field trial: it was referred to as such, and the 100 km^2^ triplets were analogous to fields in crop research undergoing experimental interventions. Indeed, the practice of randomising experimental conditions was developed by R.A. Fisher during his time at the Rothamsted Agricultural Station (Hall 2007; Berry 2015). However, instead of studying a single (domesticated) plant which (mostly) stays in one place, the RBCT was trying to understand the dynamic interactions of several very different kinds of organism: cattle, badgers, the bacterium *M.bovis*, and people.

Instead, I argue that the RBCT can be better understood as an experiment designed in the research traditions of ecology and ethology, which were also influenced by R.A. Fisher’s work—on experimental design, but also on genetics and evolution. These fields have developed research practices for building reliable knowledge about wild organisms and environments, which can be far more difficult to control and manipulate than those encountered in agriculture. While Lord Krebs is now best known as a science and policy adviser, his primary scientific expertise is in the field of behavioural ecology (e.g. Krebs and Davies 1978–2009). He is closely connected with British evolutionary biology networks, having completed his graduate work at Oxford in Niko Tinbergen’s Animal Behaviour Research Group, and is a member of the ‘Silwood Circle’ of Imperial College London, an influential grouping of British ecologists (Gay 2013).^8^ Both Krebs’ review team and the ISG maintained these connections by including members of the Oxford and Silwood groups, by working closely with MAFF/Defra’s own field ecologists, and by continuing a longstanding Silwood tradition of interdisciplinary, policy oriented, collaborative research (Gay 2013). As the name suggests, behavioural ecology integrates ecological and evolutionary perspectives with ethology, integrating laboratory based research methods with field observations, either by making the lab more like the field, or the field more like the lab (Beale 2009; Burkhardt 1999). Careful comparison of several interventions with a control and the usage of randomisation are both highly characteristic of behavioural ecology experiments, as is the employment of sophisticated mathematical modelling, and techniques such as cost–benefit analysis. When understood as behavioural ecology, the inclusion of economics in the RBCT makes sense, as does the deliberate enrolment of multiple disciplines and the early awareness of the possibilities of perturbation.

While MAFF’s earlier research programme had also involved multidisciplinary collaborations, including with ecologists, this work had been primarily directed by government field veterinarians and veterinary scientists, who followed the disease by mapping outbreaks, correlational studies, pathological and bacteriological investigation, and treatment-like interventions. This style of research fits into traditions of field epidemiology and veterinary research (Amsterdamska 2005; Steere-Williams 2015; Woods 2014), and involves less experimental and more exploratory, correlational modes of knowledge building than those underlying ecology. Unlike the RBCT, this earlier work contributed to and drew upon the professional expertise of veterinarians within government, which includes longstanding partnerships with farmers and industry (Enticott and Wilkinson 2013; Woods 2011). The arguments of Krebs and the ISG were therefore aimed at creating a break from this earlier body of research, and convincing policymakers to support their experimental approach instead. They took full advantage of what Martin Edwards has described as “the rhetoric of control” (2007, p. 39) in earlier debates over the validity of RCTs, positioning their ideas and findings as more scientific (and therefore more authoritative) than what had gone before. While Krebs et al. (1997, p. 95) had stressed that farmers should be closely involved with the proposed experiment, it is clear from the ISG’s reports that they struggled with this. In part, this was due to the unpopular suspension of badger culling across the country once the experiment had started, but the ISG’s usage of terms such as ‘non-compliance’ and ‘interference’ (ISG 1998, p. 6) to refer to the behaviour of farmers, landowners and other members of the public suggests that this was far from a participatory relationship. This can account for the persistence of critiques of the RBCT from veterinary associations, veterinary epidemiologists and farmers. While the two sides could be broadly characterised as the ‘veterinary’ and ‘ecological’ approaches, as we have seen both have involved coalitions of actors from multiple disciplines. As such, the conflict can perhaps be better understood as one between two different “ways of knowing” about the problem of badgers and bTB (Pickstone 2001).

While ecological, agricultural, epidemiological and veterinary research all meet the challenge of building knowledge about the world outside of the laboratory, each strikes a different balance between the reliability of experiment and the validity of field observation (Burkhardt 1999; Kohler 2002; Shapin 1998). These differences are key to understanding why the results of the RBCT continue to be interpreted in such diverse ways over 5 years after the ISG published their final report. Amanda Rees (2009) has argued that field science is subject to an extended version of ‘the experimenter’s regress’ (Collins 1985/1992). Any scientific knowledge claim relies upon a certain tacit level of agreement and trust in the conditions under which the experiment was conducted. In field science these will always be much more variable, and so opponents of a particular claim have much more scope to find potential flaws in the research design. Rees argues that in fields such as primatology, a ‘core-set’ of scientists willing to trust one another’s testimony prevents this process from continuing indefinitely. However, the RBCT was conducted by one core-set in order to supersede another as legitimate experts in animal disease policy, who in turn have contested its findings by pointing out flaws in the design and implementation of the experiment in the field. This was exacerbated by the scale of the RCBT, which compared to most field trials in the agricultural and ecological sciences involved a huge amount of time, space, cost, political investment, and sheer ambition. While ‘big science’ is usually used to describe high technology, high budget endeavours such the Human Genome Project (Davies et al. 2013), the RBCT can be considered as a ‘big science’ form of field research. The sheer scale of the experiment certainly increased the opportunity for events such as the FMD outbreak to disrupt the original research design, opening the findings to critique.

While most field science tends to happen in relatively inaccessible places such as field stations or wilderness environments, the RBCT happened ‘in public’ (Gregory and Miller 1998). It was conducted across the British countryside, on populated, farmed and accessible land, in the midst of a longstanding controversy, and under constant scrutiny from media, political and public actors. Bonneuil et al’s (2008) study of the French public controversy over GM crop trials discusses how these experiments were designed as an expert endeavour, but became publicly constructed as “an intrusion in the social space, which had to be negotiated with actors from that space” (p. 1). This fuelled the controversy, enrolling further actors, some of whom took direct action by destroying crop trials. The RBCT was similarly disrupted by the direct action of anti-cull protestors: ironically this has made it easier for pro-cull actors to critique its experimental conditions and counter the case *against* culling. Such cases reveal a further dimension to the ‘problem of place’ (Burkhardt 1999) in field science. Not only does the place where research is done shape scientific practices, it also shapes the relationships built between scientists, other people, and what those people know about that place. This is important for relationships between scientists and those nearby, such as farmers and local communities (Wynne 1992), but also in wider public and policy conflicts about the aims, purpose, framing and interpretation of field science (Berry 2015; Harwood 2012).

Given the complexity of the badger/bTB problem, the ISG’s research can be regarded in and of itself as a remarkable achievement, one which continues to have a profound influence on scientific, public and policy debates (e.g. Godfray et al. 2013). While the RBCT drew upon several research traditions, the challenges it faced in practice transformed it into a unique experiment: one which was forced to negotiate considerable complexities and unanticipated problems, arising in part from the actions of the people and animals involved. Histories of field science show us that these problems and the improvisations made to deal with them are not that unusual, albeit unanticipated and particularly severe in this case. From the beginning of this process the ongoing controversy has turned upon a shared failure to acknowledge the contingencies and uncertainties of ‘the science’ of badgers and bTB. While Krebs and the ISG drew upon the rhetoric of authoritative science to argue that they could resolve a chronic policy problem, this self-same rhetoric has made it much easier to undermine and contest their findings. At the same time the critics of the RBCT highlight these uncertainties to argue that the experiment is flawed: this elides the clear difficulties involved in producing authoritative, incontestable scientific knowledge about this issue in the first place. Ultimately, the now-routine invocation of ‘the science’ in support of arguments both for and against badger culling ‘cannot meaningfully contribute’ to solving the UK’s bTB problem. To conclude, while the case of the RBCT does not mean that RCTs can never constructively feed into policy, it does suggest that advocates of this position should direct their efforts at anticipating and acknowledging the complexities of the knowledge produced for such purposes, and be much more aware of the dangers of expecting policy on *any* topic to be straightforwardly ‘evidence based’.

## Footnotes

1 It is not possible to fully explore this earlier research programme in this article for reasons of space: however it is a key focus of the author’s ongoing research, and as such will be examined in detail in further publications.

2 Between them Grant (2009), Spencer (2011) and Barkham (2014) provide useful, if brief overviews of the sequence of events from the early 1970 s until 2010.

3 The existence of transmission links between cattle and badgers continued to be disputed until the end of the RBCT, and their importance for TB infections in cattle continues to be contested by some actors.

4 Cunningham also received the first report of the ISG, alongside overseeing the early stages of the post-BSE reform of MAFF. He then moved to the Cabinet Office, where among other things he was a core advocate of Labour’s reform and ‘evidence based policy’ agendas (Cabinet Office 1999).

5 For the benefit of readers outside of the UK, Prime Minister’s Questions is a weekly session in the UK Parliament where the PM is directly questioned by Opposition MPs. By longstanding tradition and reputation, it is a notoriously oppositional and rowdy event.

6 Recently published correspondence between HRH the Prince of Wales and the then PM Tony Blair now suggests that this inference was indeed correct, and Blair was in favour of a culling policy (Cabinet Office 2015).

7 While several other trials of badger culling of various sorts have been undertaken in the UK and Republic of Ireland both before and since the RBCT, none of these have involved this specific research design.

8 The Silwood group also includes former UK Chief Scientists Bob May and John Beddington, and the epidemiologist Roy Anderson. As well as undertaking the epidemiological modelling guiding UK policy on the 2001 FMD outbreak, Anderson was a member of Krebs’ 1997 review group. The Oxford Animal Behaviour Research Group has at various times also included Richard Dawkins, Desmond Morris, and badger specialists Hans Kruuk and David Macdonald.
